# 

**Published:** 1878-01-20

**Authors:** 


### INDEX.

A

Abingdon Academy of Medicine........ 30
Abortion, retained placenta in........ 149
Abscesses, sulphuric acid........ 391
Acetic acid, injection in carcinoma........ 391
Acid, carbolic, antidote to........ 315
Acid, carbolic, perfumed, pres. for........ 315
Acid, boracic, in skin diseases........ 153
Acid, hydrobromic, cerebro spinal meningitis........ 138
Acid, salicylic, vehicle for........ 250
Acid, salicylic, for children........ 250
Acid, salicylic, in rheumatism........ 22
Acetate of lead in granular conjunctivitis........ 343
Acne rosacea, treatment of........ 244
Aconite, comparison of with opium& belladonna 384
Aconite, tincture........ 348
Action of pilocarpin on the eye........ 83
A curious discovery........ 84
Acute catarrh of the middle ear........ 82
Addison's disease, a case of........ 6
Adhesive plaster, a new........ 158
Adolphus Dr. Joseph, on preparation of pepsin.. 369
Ague, carbolic acid for........ 348
Ailanthus glandulosa,........ 337
Alcoholism, chronic, oxide of zinc in........ 259
Alcoholism, treatment of........ 186
Allopath vs. any other "Path"........ 93
Alport, Dr. Frank, on treatment of fevers........ 103
Alimentation, rectal........ 42
Alum in aural discharges........ 179
Amorrhœa........ 396
American Gum Arabic........ 56
American Holly........ 348
American Med. Ass. officers of........ 256
Amyl, nitrate of, in asphixia........ 181
Amputation by ligature........ 85
Anemia, tonic in........ 316
Anesthetics, employment of in labor........ 17
Anesthetics, in puerperal convulsions........ 309
Anesthetics, local........ 340
Anesthetics, unusual action of........ 44
Anatomy, pathological, recent progress in........ 306
Anderson, Dr. S. H., case in practice........ 321
Anderson, Dr. S. H., on hemorrhage from tooth extraction........ 163
Anderson, Dr. S. H., note on capillary bronchitis 281
Anderson, L. B., on poisoning with tainted meat 3
Anginose affections, oil of amber in........ 336
Antagonism of therapeutic agents........ 131
Antagonism of belladonna and opium........ 20
Anti-colic mixture........ 220
Anti-dyspeptic powder, McCall's........ 218
Antidote to arsenic........7— 316
Antidote to carbolic acid........ 315
Antidote to strychnia, coffee as........ 20
Anti-fat........ 222
Anti-insects........ 158
Anti-rheumatism pills........ 396
Ants, armies of........ 189
Aneurism treated by use of Esmarch bandage..... 171
Aperient, saline........ 157
Apthous ulceration........ 219
Arm presentations........ 209
Arm presentations, turning in........ 372
Aromatic syrup of liquorice........ 16
Arsenic in skin diseases........124—155— 313
Arsenic in bismuth........ 219
Arsenic in mania........ 158
Arsenic in the treatment of malignant tumors... 151
Arsenic, rules for its therapeutic use........ 203
Arsenic, treatment of bromide rash with........ 240
Arsenic, bromide of, in nervous diseases........ 247
Arsenical paste........ 66
Arsenical poisoning, dialized iron in........ 7
Artificial larynx........ 189
Asparagus root........ 348
Asphyxia, nitrate of antimony in........ 181
Asthma, iodide of ethyl in........ 113
Asthma, remedy for........ 220
A strange case........ 180
Atlanta Medico-Chirurgical Association........48— 87
Atropia hypodermically........ 157
Aural discharge, alum in........ 179
Auscultation, telephonic........ 92

B

Bacteria, and what will kill them........ 81
Ballooning spiders........ 349
Bandage, strong elastic, for ulcers........ 176
Bath of decreasing temperature in typhoid fever 9
Baths, warm, in tetanus........ 185
Bedford Springs iron and alum mass in yellow fever........ 290
Beer and suicide........ 181
Belladonna, antagonism of to opium........ 20
Belladonna, comparison of with opium and aconite........ 384
Belladonna in collapse........ 121
Belladonna in dysentery........ 118
Bicarb. soda........ 393
Bismuth, arsenic in........ 219
Bismuth in treatment of prolapse of the rectum.. 155
Bitters........ 396
Bladder, washing out the........ 21
Blister, extemporaneous........ 185
Blood, influence of iron in food on........ 84
Blood, the in diphtheria........ 86
Bone and nerve liniment........ 58
Book notices........31, 64, 94, 128, 191, 223, 255, 287, 319, 352
Boracic acid in skin diseases........ 153
Boracic acid ointment........ 187
Boring, Dr. Jno. M., on cauterizing os for nausea of pregnancy........ 302
Brain, duality of the........ 134
Brain, minie ball in the, 15 years........ 323
Breasts, management of, without nursing........ 339
Breech presentations........ 11
Bright s Disease, use of sulphate of cinchonidia in 198
Bromide of potassium for chills........ 347
Bromide rash, treatment of with arsenic........ 240
Bronchitis, capillary........ 281
Bronchitis, capillary, of children, wine in........ 343
Bronchitis, strychnia in........ 121
Bronchitis, prescription for........ 27
Brute ceremonies........ 284
Bryonia and Drasera in whooping-cough........ 309
Burns, bicarb soda for........ 121
Burns, treatment of........ 122

C

Cæsarian section after death........ 311
Caffine in cephalalgia........ 346
Calcium chloride in chronic diarrhœa........ 386
Calcium chloride in phthisis........ 386
Calcium chloride in tabes mesinterica........ 386
Calisaya and Bismuth, mixture of........ 283
Calomel........ 340
Calomel, alteration of........ 347
Calomel, a substitute for........ 283
Calomel, saccharated........ 59
Caffine, in cephalalgia........ 346
Campbell, Dr. E. M., of Va., death of........ 224
Camphynel........ [illegible]

Cancer, Esmarch on ... 177
Cancer of the breast, treatment of ... 270
Cancer, treatment of by pressure ... 248
Carbolate of Soda, in affections of respiratory passages ... 115
Carbolic Acid, antidote to ... 315
Carbolic Acid, perfumed ... 315
Carbolic acid, for ague ... 348
Carbuncle, ... 180
Carbuncle, Guerin's treatment for ... 121
Carcinoma, acetic acid injection in ... 391
Carminative, Dalby's ... 60
Case of successful transfusion ... 77
Cases of varicose veins ... 80
Caston, Dr. John, case of Hysteria epileptica ... 37
Castor oil, emulsion ... 252
Castor oil seeds, poisonous, dose of ... 20
Catarrh, acute, of middle ear ... 82
Catarrh, Bronchial, pres for ... 348
Catarrh, cure, Weatherly's ... 157
Catarrh and Laryngeal affections ... 26
Catarrh, yerba ruma in ... 124
Catarrh snuff ... 396
Cauterization of cervix, for vomiting in pregnancy ... 302
Cauterization of Nævi, Thirsch's method of ... 249
Cement, a durable ... 188
Cephalalgia, Caffine in ... 346
Cerebral hemorrhage, prognosis in ... 346
Chalk and mercury, when dangerous ... 219
Chalk mixture, improved ... 188
Cheap tonic ... 26
Cherry laurel water, for ophthalmia in infant s... 120
Chicken pox, cure for ... 156
Children, diarrhœas of ... 372
Children, the medical treatment of ... 213
Chilblains, cure for ... 59
Chills, chronic, treatment of ... 313, 347
Chills, intermittent, cure for ... 156
Chloral hydrate ... 23, 78
Chloral hydrate, used per rectum ... 327
Chloral hydrate used with opium ... 33
Chloral, by enema ... 151
Chloral, dangerous in delirium tremens ... 188
Chloral, in dysentery ... 247
Chloral, hypodermic use of in cholera ... 283
Chloral and glycerine in constipation ... 249
Chloral and opiates, danger of using alternately.. 68
Chlorate of potash, in diarrhœa ... 177
Chlorate of potash, in Ulcers ... 380
Chlorodyn, ... 316
Chloroform, death from ... 390
Chloroform, in labor, ... 238, 249
Chloroform and morphia combined, use of ... 16
Chloroform hallucination ... 83
Chloroform hallucination and alleged rape ... 17
Chloroform, spirit of ... 220
Chlorymal, ... 395
Cholagogues, ... 298
Cholera, hypodermic use of chloral in ... 283
Cholera infantum ... 235
Cholera, prize offered for cure of ... 119
Chorea, zinc for ... 315
Chronic ague, pres. for ... 59
Chronic gonorrhœa, treatment of ... 52
Chronic intermittens, ... 274
Churchills tinc. of Iodine ... 60, 182
Cinchonia ... 90
Cinchonia alkaloid ... 318
Cinchonidia, sulphate of, in Brights disease, ... 198
Cincho-quinine, properties of ... 126
Citrate of iron quinia and strychna ... 282
Clark, Dr. J. E., on breech presentation ... 11
Coal tar, alchoholic solution of in comminuted fracture ... 382
Coca erythroxilon ... 255
Codliver oil, idoformized ... 395
Codliver oil, with hypophosphite and whisky... 187
Coffee as an antidote to strychnia ... 20
Colica pictonum ... 130
Colic, pres. for ... 220
Collodion bandage, in umbilical hernia ... 334
Collodion paints, ... 25
Comminuted fracture of the leg, treated without splints ... 68
Compression, trephining for ... 150
Condition powder, for horses ... 58
Congenital malformation ... 375
Congenital phimosis, with adherent prepuce ... 199
Congestion, use of opium in ... 35
Conium, ... 22
Constipation, remedy for ... 91, 220, 249
Constipation, cascara, sagrado in ... 334
Consumption, koumiss, glycerine, and creesote in 152
Contraction of the fingers ... 338
Copper poisoning, case of ... 47
Cordial, Godfrey's ... 58
Cordial, neutralizing.., ... 187
Corneal ulcers ... 40
Cornstalk pith as a uterine tent ... 260
Corrosive sublimate, hypodermically ... 157
Corrosive sublimate, in dysentery ... 101
Costiveness, permanent cure for ... 24
Cota bark, ... 337
Cota bark, in diarrhœa and rheumatism ... 57
Cough mixture, ... 251
Cough syrup, ... 283
Coxitis, hemorrhagic, cured by puncture ... 248
Cramp, night ... 35
Creosote in ulcers ... 158
Croup, membranous ... 27
Croup, treatment of ... 86, 343
Cure, instantaneous, of hydrocele.. ... 56
Cyanide of zinc for rheumatism ... 59

D

Datura as a medriatic ... 217
Dermalgia, from quinine, ... 383
Delerium tremens ... 26, 185
Dentistry, new departure in ... 287
Dentition, fever of, remedies in ... 300
Diabetes and eczema......184, remedy for 121, 60, 336
Dialized iron ... 29
Diarrhœa coliquitive, ... 251, 314, 124
Diarrhœa, in children, ... 372, 188, 155
Diarrhœa, cota bark in ... 57
Diarrhœa, prescription for 156, oxide of zinc in..... 390
Diarrhœa, chronic, calcium chloride in ... 386
Diarrhœa, prescription for ... 177, 156
Diarrhœa, chronic prescription for ... 282
Diarrhœa, mixture, Gould's ... 282
Diphtheria, ... 87, 251, 330
Diphtheria, paralysis of ... 24
Dislocation of shoulder, new method ... 54
Disgust, philosophically considered ... 125
Doctors, testimony in behalf of ... 183
Dog fennel ... 348
Doyle, Dr. O. M., of Ga., gun shot wound of the brain ... 323
Dogs, strychnia poison in ... 219
Draper, Dr. John, as a man of science ... 397
Dressing stump, new method of ... 181
Duty of Doctors to the dying ... 69
Duality of the brain ... 134
Dynamite ... 116, 253
Dyalized iron, (vs. arsenic) ... 7
Dyspepsia ... 1
Dyspepsia, flatulent ... 389
Dyspepsia, malarial ... 282
Dyspepsia, at Demilt Dispensary ... 394
Dysentery, corrosive sublimate in ... 101
Dysentery, Iodi form in, ... 389
Dysentery, belladona in ... 118
Dysentery, chloral in ... 247
Dysentery, chronic, nitrate silver in ... 248
Dysentery, morphia injections in ... 270
Dysentery, ailanthus glandulosa in ... 282
Dysentery mixture, Hope's ... 188
Dysmenorrhœa ... 220, 187, 237
Dysmenorrhœa, complicating eczema ... 178
Dysmenorrhœa, Pulsatilla, in ... 178

E

Eczema, glycerole of subacetate of lead in ... 341
Eczema capitis, ointment for ... 348
Eczema, its connection with diabetes ... 184
Edison's phonograph ... 28
Edison's laboratory and experiments ... 397
Electric light ... 61, 125, 317, 279
Elephantiasis of the penis ... 55
Electro-puncture for hydrocele ... 154
Elixir guarano ... 62
Elixir valerianate of ammonia ... 283
Elixir of quinia and iron ... 282

Elixir of valerian........ 60
Emulsion of cod liver oil........ 187
Emulation........ 398
Envy........ 63
Epistaxis........8, 82
Epithelioma........287, 388
Epilepsy, treatment of........ 117
Ergot, effect on circulation........ 23
Ergot, death from........ 339
Ergot in diabetes........ 121
Ergot hypodermically........ 157
Ergot in keloid........ 333
Ergot in polyuria........ 152
Ergot, treatment of cystic tumor with........ 161
Ergot, action of on uterus........ 179
Ergot in enlarged prostate........ 239
Eruption of poison oak........ 154
Erysipelas, silicate of soda in........ 53
Esmarch's bandage in aneurism........ 171
Ethyl iodide in asthma........ 113
Etiology of infectious diseases........ 61
Eucalyptus, extract of as a local anæsthetic........ 185
Evaporating lotion........ 91
Eve, Dr. Paul F., tribute to........ 63
Evisceration without instruments........ 324
Evolution........ 397
Exophthalmia treated by galvanization........ 182
Expert, the medical........ 172
Extract of malt as an emulsifier........ 118
Eye, action of pilocarpin on........ 83
Eye-water, excellent........ 91
Eye, diseases of........ 208
Eye diseases, oleate of mercury in........ 248

F

Favus, treatment of........ 182
Felons, cure for........ 314
Femur, fracture of........164, 185
Feeding per rectum........ 120
Fever, intermittent, cinchonidia in........ 198
Fever, intermittent, common salt for........ 315
Fever, intermittent, iodine for........ 315
Fever, intermittent, treatment of........ 249
Fever, intermittent, with complications........ 281
Fever, malarial and yellow........ 271
Fever of dentition........ 300
Fever, remedies for........ 305
Fever, typhoid, baths in........ 9
Fever, typhoid of children........ 230
Fever, typhoid, turpentine........60, 300
Fever, typho-malarial........ 250
Fevers, management of in Germany........ 229
Fevers, picrate of ammonium in........ 89
Fevers, use of cold and heat in........ 103
Fibroid tumors of the uterus........ 390
Fire, singular........ 221
Fissure of the rectum........ 38
Flatulent dyspepsia........ 389
Flies, to keep off........ 396
Follicular stomatitis........ 57
Food with iron, effect of........ 84
Ford, Dr. DeSaussure, on tar-water........ 257
Fowler, Dr. R., of Texas—a query........ 250
Fracture, compound comminuted of ankle........ 382
Freezing process, a new........ 189
Frost-bite........ 396
Frozen air........ 253
Fucus vesiculosus........ 223

G

Gallium, the new metal........ 284
Galvanization in exophthalmia........ 182
Galvanism in uterine fibroids........ 303
Ganglion, treatment of........ 280
Genito-urinary surgery........ 335
Gentian, wine of........ 220
Germs, the latest concerning........ 153
Glazener, balsam of fir in wounds........ 394
Goiter........ 99
Goldsmith, Dr. W. T., on uterine tents........ 260
Gonorrhea, chronic........ 52
Gonorrhea, pathology and treatment........ 136
Gonorrhea, injection for........ 187
Goss, Dr. I. J. M., on metorrhagia........ 5
Gout, saracena purpura in........ 185
Graves' disease........ 50
Greene, Dr. W. A., yellow fever........ 289
Greene, extract of malt, &c........ 225
Greenley, Dr. T. B., on hypophosphite of soda........ 129
Greenley on yellow fever........ 289
Grindelia robusta in whooping-cough........ 119
Guarano, elixir........ 62
Guiacum in quinsy........ 124
Gum arabic, American........ 56
Gurjon balsam in gonorrhea........ 79
Gutta percha tissue........ 57

H

Hair dye black without silver........ 59
Hallucination, chloroform, alleged rape........17, 83
Hardman, Dr. L. G., of Ga., on congenital malformation........375
Head, injuries of........211
Heart disease, organic, remedy for........251
Hemorrhage, cerebral, prognosis in........346
Hemorrhage after delivery, transfusion for........ 77
Hemorrhage, cinnamon in........252
Hemorrhage, obstinate, from extracting tooth........163
Hemorrhage, post partum........ 91
Hemorrhage post partum, perchloride of iron in...217
Hemorrhage, uterine........ 5
Hemorrhages........277
Hemorrhagic coxitis, case of........248
Hemorrhoids........54, 124
Hemorrhoids, bismuth for........155
Hemorrhoids, internal, glycerin for........116
Hemorrhoids, treatment of........ 65
Herndon, Dr. Z. B., on fracture of the leg........68
Hernia, strangulated, treated by hypodermic injection........268
Hernia, umbilical........ 56
Hernia, umbilical, case of........234
Hernia, umbilical, collodion bandage in........344
Hiccough cured by compression........336
Hiccough treated by pilocarpin........247
Hiccough treated with jaborandi........249
Hoarseness, borax and nit. potass. in........120
Hobbs, Dr. A. G., on pneumonia........ 90
Hobbs, Dr. A. G., on turning in arm presentations 372
Hæmoptysis........154
Hope's dysentery mixture........188
How to introduce the hypodermic needle........ 86
Hughes, Dr. C. H., essay on rheumatism........ 72
Hutchins, Dr. R. E., remedy for worms........ 91
Hydrargyrum cum creta, when dangerous........219
Hydrate chloral........23, 78
Hydrate chloral, unusual energy with opium........ 33
Hydrobromic acid in cerebro-spinal meningitis....147
Hydrocele, instantaneous cure of........56, 154
Hydrocele of infants, nitrate of ammonia for........217
Hypodermic injection of atropia........157
Hypodermic injection of corrosive sublimate........157
Hypodermic injection of ergotin........157
Hypodermic injection of morphia in dysentery.....270
Hypodermic injection of morph., warning against 205
Hypodermic needle, how to introduce........ 86
Hypophosphite of zinc........140
Hypophosphite of soda, uses of........129
Hysteria, combination for........ 59
Hysteria epileptica, with complications........ 37
Hysteria in a boy........245
Hysteria, phosphide of zinc in........ 57
Hysteria, treatment of........ 14

I

Ice water treatment of croup,........ 86
Illusions........ 349
Impotence........ 145
Impotence and spermatorrhœa........ 395
Infants, diarrhœa in........ 155
Inflammable liquids, danger from........ 117
Inflammation of the Os, or vaginismus........ 25
Influence of iron mixed with food on the blood........ 84
Injection intra-venous, of milk........ 204
Injection, inter uterine, without use of speculum ... 25
Injuries of the head........ 211
Ink, blue black........ 396
Insects........ 252
Instantaneous cure of hydrocele........ 56
Instrumental labor........ 70
Inter-uterine injections without speculum........ 25
Intermittents, quartan, cure for........ 156

Intermittents, chronic.... 274
Interm ttents, with conjestion of the liver.... 281
Intermittents, with irritable stomach in.... 281
Intertrego in children.... 246, 391
Intestine irrigation of large.... 12
Intestinal gas.... 389
Iodia in tubercular meningitis.... 193
Iodine, purpura....138 23
Iodine, tinc. absorption by the skin.... 153
Iodine, how to deprive of its stain.... 176
Iodine, Churchill's tinc. of.... 183
Iodine in fever.... 305, 315
Iodinized milk, uses of.... 233
Iodoform in dysentery....174
Iodoform ointment.... 340
Iodoform.... 112
Iodoform, for syphilitic sores.... 385
Iodoformized codliver oil.... 395
Iron, bichloride, in shingles.... 184

J

Jaborandi in hiccough.... 249
Jaundice.... 172
Japan, prevalence of our diseases in.... 253

K

Keloid Tumor, ergot injection for.... 333
Kerosene liniment.... 27
Kilpatrick, Dr. A. R., on chloral and opiates.... 68
King, Dr. F. on dyspepsia.... 1
Koumiss, glycerine and creosote in consumption.... 152
Koumiss, formula for.... 178

L

Labor, anaesthesia in....17, 238, 249
Labor instrumental.... 70
Labor, aids to.... 307
Labor, protracted.... 19
Lactation, excessive.... 57
Lactopeptine.... 29
Lactopeptine, in infantile diarrhœa.... 252
Laryngeal affections and catarrh.... 26
Larynx, excission of.... 117
Larynx, artificial.... 189
Lead colic.... 334
Leucorrhœa.... 252
Ligature, amputation by.... 85
Liniment, kerosene.... 27
Lincecum, Dr. L. Q., on hemorrhoids.... 65
Liquefaction of gases.... 61
Liquorice, aromatic syrup of.... 16
Liquor, ferri, sesquichloride, injected for croup.... 343
Lister's antiseptic method.... 51
Litmus paper, substitute for.... 60
Liver, enlarged.... 49
Liver pill.... 49
Loving, Dr. A. B., opium in congestion.... 35
Loving, Dr. A B., hyposulphite of soda.... 156
Long, Dr. Crawford W. death of.... 255
Lotion, evaporating.... 91
Louisiana Med. Association of.... 127
Loefland's Leibig's food.... 225
Lupus.... 246
Lyon, Dr. A. A., on wound of penis.... 227

M

Malt, extract....344, 225
Malformation, congenital.... 375
Mammary al s ess.... 310
Manganese, sulphate of for calomel.... 283
Mania, arsenic in.... 158
Mate, or Paraguay tea.... 216
Maternal impressions.... 174
McDonald, Dr. G., instrumental labor.... 70
McCall's antidyspeptic powder.... 218
Measurements, approximate.... 157
Measles.... 128
Meat juice.... 337
Med. Association of Ga., (transactions of 223).... 144
Med. Asso., disaffection in.... 159
Medical Society of Arkansas.... 160
Medico-Chirurgical Society, Atlanta.... 48
Medical progress in 1877.... 61
Medical Aphorisms.... 113
Medical Literature and Associations.... 126
Me i o--legal evidence.... 242
Membranous croup,.... 27
Membranes, early rupture of.... 186, 27, 97
Meningitis, cerebro-spinal.... 138
Meningitis, tubercular, diagnosis by smell.... 153
Meningitis, tubercular, iodia in.... 193
Menorrhagia....124, 60
Metroragia, egotin in.... 187
Menstruation and ovulation.... 117
Metric system....207, 280
Midwifery practice in.... 100
Milk diet in the puerperal state.... 320
Microscopical observations in yellow fever,.... 373
Microphone.... 221
Milk iodinized, uses of.... 233
Milk, salt in for babies.... 235
Moon.... 317
Moles, removal of.... 121, 387
Morphia hypodermically, caution with.... 205
Morphia, muriate.... 348
Morphia, muriate of, injections for hernia.... 268
Morphological changes in syphilitic blood.... 247
Mothers milk, substitute for....251, 243
Muriate of Calcium in tuberculosis.... 182
Mucous inflammation, œnothera biennis in.... 46
Mustard baths in pneumonia of children.... 340

N

Nauseating effects of opium, remedy for.... 311
Nævus removal of.... 387
Newton, Dr. O. E., how to give podophyllin.... 24
Newton, Dr O. E., on removal of placenta.... 24
Newton, Dr. O. E., practical notes....58, 186
Newton, Dr. O. E., on painters colic.... 130
New method of reducing dislocations of shoulder... 54
Needle, hypodermic, how to introduce.... 86
Nervine.... 348
Nervine and antispasmodic.... 252
Neuralgia, remedy for.... 158
Neuralgia, caused by uterine disease.... 376
Nervous diseases, bromide of arsenic in.... 217
Nervousness and tobacco.... 219
Nerve impulses, velocity of.... 284
Neutralizing cordial.... 187
Night cramp.... 25
Night sweats,....124, 156
Nipples, sore, remedy for....27, 201
Nipples, cracked treatment of.... 116
Nipple wash, Dr. Atlee's.... 188
Nitro-muriatic acid.... 116
Nitrate of silver ointment.... 388
Nose, to remove fungus-growths from.... 316
Nocturnal pains.... 157
Nursing sore-mouth.... 390

O

Obstetrical operations, remarks on.... 146
Oenothora biennis in mucous inflammations.... 46
Oil of amber in anginose affections.... 336
Ointment, iodoform.... 340
Ointment, boracic acid.... 187
Onychia maligna, cure for....283, 334
Opium, use of in congestion.... 35
Opium, antagonism of belladonna.... 20
Opium nausea, remedy for.... 311
Opium, deodorized tincture of.... 348
Opium, comparison of with belladonna and aconite 384
Opium habit.... 273
Ophthalmia, purulent of infants,.... 120
Os uteri, caution as to.... 250
Ovarian tumors.... 27
Ovarian dyspepsia.... 393
Ovariotomy,.... 110
Ovariotomy,.... 182
Ovariotomy, superseded.... 389
Oxalate of cerium and caffien as remedies for opium nausea.... 311
Ozaena, treatment of.... 18

P

Paralysis of diphtheria.... 24
Paralysis, pseudo-hypertrophic.... 25
Paralysis, from reflex action and hemorrhoids, bismuth in.... 173
Painters colic.... 130
Pains, nocturnal.... 157

Paraguay tea.... 216
Pancreas the, in Diabetes.... 83
Pancreatic fluid.... 393
Palpitations nervous, remedy for.... 251
Penis, elephantisis of.... 55
Penis, laceration of.... 2-7
Perineum, laceration of.... 122
Penthorum sedoidis.... 157
Pepsin, preparation and uses.... 369
Philadelphia, mortality in.... 27
Phonograph, Edison's.... 28
Phosphide of zinc, in hysteria.... 57
Pharmceutical Ass. of Ga.... 128
Phthisis.... 304
Phthisis, tapping a vomica in.... 310
Phthisis, tonic in.... 252
Phthisis, calcium chloride in.... 386
Photographing with lightning.... 317
Phimosis, congenital, case of and treatment.... 322
Phimosis, congenital, with adherent prepuce.... 199
Phosphorus in sciatica.... 245
Piles treatment of.... 175
Piles,, internal, operative treatment of.... 183
Pill podophyllin.... 59
Pill Liver.... 49
Pill anti-rheumatic.... 396
Pilocarpine, effect on the eye.... 83
Pilocarpine, for hiccough.... 247
Picrate of ammonium in favus.... 89
Pimple cerate.... 396
Placenta, how to remove.... 24
Placenta, retained in abortion.... 149
Placenta previa, treatment of.... 169
Placenta previa, prophylactic treatment of.... 215
Plaster, adhesive, a new.... 158
Pneumonia of children, mustard baths in.... 340
Pneumonia, treatment of.... 215
Pneumonia, veratrum viridi in.... 90
Poisoning with tainted meat.... 3
Poisoning arsenical, case and treatment of.... 7
Poison oaks, ivy, etc., remedy for poisoning from.... 154
Podolphyllin, how to give.... 24
Podophyllin pill.... 59
Posey, Dr. W. S., on rupture of membranes.... 97
Potassium, bromide, in vomiting of pregnancy.... 107
Post-partum hemorrhage, case of.... 111
Post-partum hemorrrhage, vinegar in.... 312
Post-partum hemorrhage, case of.... 32i
Polypus on the uterus.... 115
Polypus fibroid, of the vagina.... 155
Porr's operation, successful case of.... 304
Polymicroscope.... 317
Populus trunuloids of American asper.... 348
Polyuria, treated with ergot of rye.... 152
Pond's American sphygmog aph.... 262
Protracted labor.... 19
Pruitus vulvæ.... 23, 123, 262, 331, 393
Princeton Review.... 287
Prolapse of the rectum and hemorrhoids, bismuth in 155
Pregnancy and suckling.... 184
Pregnancy, diagnosis of.... 220
Prostate, enlarged.... 239
Professional taxes.... 287
Psoriasis vulgaris ambulatory treatment of.... 306
Ptyriasis versicolor.... 391
Puerperal fever preventive, treatment of.... 106
Puerperal convulsions, anaesthetics in.... 309
Purpura, iodic.... 138
Pulvis paregoricus.... 316
Putrefaction.... 189
Purgative powder.... 157

Q

Quartan intermittens, remedy for.... 150
Quackery.... 254
Quinia, substitutes for.... 19
Quinia, to conceal taste of.... 336
Quinia, how to dispense.... 218
Quinine, for hypodermic use.... 27
Quinine, dermalgia, etc., from.... 383
Quinine theory of the action of.... 246
Quinine, urticaria from.... 53
Quinine, a vehicle for.... 310

R

Rats, to kill.... 89
Rectum, fissure of.... 38
Rectum, prolapse of, bismuth in.... 155
Rectal alimentation.... 42, 120
Retinal image persistence of.... 125
Respiratory passages nervous affection of.... 184
Rheumatism, coto bark in.... 57
Rheumatism, cyanide of zinc in.... 59
Rheumatism, treatment of.... 20
Rheumatic tonic.... 188
Rhubarb, valerianated syrup of.... 158
Rigors, remedy for.... 187
Rigid os.... 257
Rupture of membranes.... 97
Rushing, Dr. F. M., laceration of perineum.... 122

S

Saccharated calomel.... 59
Salicylic acid.... 22
Salicylic acid, vehicle for.... 250
Salycylate of sodium in diabetes.... 336
Salt, common, as a remedy for intermittent fever.... 315
Santonin.... 17
Santonin, poisoning.... 220
Sarracenia purpura, in gout.... 185
Sayre's plaster jacket in spinal disease.... 335
Scabies, oil of stavesacre in.... 184
Scalp wounds.... 158
Scarlatina, treatment of.... 84
Scarlatina, sore throat in.... 185
Scarlatina, prophylaxis of.... 179
Sciatica, treatment of.... 20
Sciatica, treated with phosphorus.... 245
Sclerotic acid, in fibroid tumors.... 390
Shields, Dr. S. S., on chloral and opium.... 33
Shingles, perchloride of iron for.... 184
Silicate of soda, in erysipelas.... 52
Silk worms, to hatch.... 284
Simple elixir.... 219
Skin diseases, boracic acid in.... 153
Skin diseases, arsenic in.... 155, 124, 313
Smart weed.... 314
Small pox, turpentine for.... 280
Smell, sense of as a means of diagnosis.... 153
Smith, Dr. E. L., duty to the dying.... 69
Smith, Dr. G. W., on treament of burns.... 122
Society medico chirurgical, Atlanta.... 48, 87, 89, 140, 172, 235, 325
Soda, carbonate of in diseases of respiratory organs 184
Soda, carbonate, in burns.... 121
Sodium ethylate, removal of moles by.... 387
Soda hyposulphite.... 129
Soda hyposulphite, in chicken pox.... 156
Sore nipples.... 27, 201
Sore throat, in scarlatina.... 185
Southern Medical College.... 400
Spermatorrhea and impotence.... 395
Spermatic Truss.... 29
Spinal curvatures.... 388
Spinal diseases, treatment of.... 335
Stammering.... 332
Stavesacre, oil of, scabies.... 184
Stibrodermic treatment in rheumatism.... 120
Stoppers, to obviate sealing of.... 220
Stricture, urethral.... 210
Stumps, new method of dressing.... 181
Subacetate of lead, glycerole of in eczema.... 341
Subcutaneous medication.... 157
Substitute for mother's milk.... 251, 243
Suggs, Dr. J. T., case reported.... 324
Suicide and beer.... 181
Strychnia.... 158
Strychnia, its action on the eye.... 217
Strychnia, as a tonic.... 338
Strychnia, coffee as an antidote to.... 20
Strychnia, in bronchitis.... 121
Strychnia in dogs, remedy for.... 219
Sulphurous acid, in pruritus vulvae.... 331
Sulphurous acid, in abscess.... 391
Sullivan, Dr. J. F., case in practice.... 46
Summer complaint, cure of.... 252, 119
Sun, the metals in.... 2[illegible]8
Sun's distance.... 28
Sun spots and terrestrial magnetism.... 92
Sun-fish and the submarine cabal.... 221
Surgical knots.... 216
Syrups officinal.... 218
Syphilis in the negro and white man contrasted.... 328
Syphilis secondary.... 123

Syphilitic rheumatism........ 27
Syphilitic sores........ 385

T

Tabes mesenterica........ 386
Tainted meat, poisoning with........ 3
Talipes-equino—varus operation for........ 276
Tannin in the vomiting of pregnancy........ 112
Tannin, as a deodorizer of iodoform........ 13
Tannin as a medical agent........ 124
Tansy in pruritus vulvae........ 262
Tape-worm, remedy for........26, 357
Tape-worm, rapid expulsion of........ 154
Tape-worm new treatment of........ 247
Tar water, antiseptic properties of........ 257
Tartarized antimony........ 387
Taxing medical men........ 351
Teeth, diseases of........300, 58
Teeth, anaesthetic in extracting........ 340
Telephone, uses of in scull fractures,........ 21
Telephonic auscultation........92, 221
Telephone in China........ 253
Telegraphing without wires,........ 125
Tetanus traumatic........ 185
Therapeutic agents, antagonism of........ 131
Thoracentisis........ 75
Tinea favosa........ 385
Tobacco, in nervousness........ 219
Tonics........26, 188, 338
Tonsilitis........ 183
Tracheotomy........202, 56
Transfusion, successful case of........ 77
Triostium perfoliatum........ 348
Trephining, for compresssion........ 150
True bravery........ 319
Tuberculosis, muriate of calcium in........ 182
Tumor, cystic, treatment with ergot........ 161
Tumors malignant, arsenic for........ 151
Tumors, ovarian, (fibroid 390)........ 27
Turpentine externally in small pox........ 280
Turpentine, in typhoid fever........ 60
Typhoid fever........ 9
Tophoid fever in children........ 230
Tying surgical knots........ 219

U

Ulcers, creosote in........ 158
Ulcers, treament with bandage........ 176
Ulcers, treatment........180, 390
Ulcer, corneal........ 19
Ulcer of the leg, chloride of zinc in........ 148
Ulcer, tubercular of the tongue........ 386
Ulceration apthous........ 219
Ulceration of the os uteri........ 19
Umbilical hernia........287, 56, 234
Urethra, dilation by urine........ 278
Urea formation........ 389
Urethra, stricture of........ 210
Urinal, new........ 233
Urticaria........ 396
Urticaria and quinine........ 56
Ustilago maidis........ 164
Uterus, action of ergot on........ 179
Uterine disease as a cause of neuralgia etc........ 376
Uterine tent with pith of cornstalk........ 260
Uterine fibroids........ 303

V

Vaccination, syphilis transmitted by........ 182
Vaccination, with horse lymph........ 116
Valerian, elixir, of........ 60
Valerianated syrup of rhubarb........ 158
Valerinate of ammonium........ 283
Valentine meat juice........ 337
Varicocele. treated by subcutaneous ligation........ 214
Varicose veins, cases of........ 80
Vasaline in pharmacy........ 347
Vermifuges........ 26
Veratrum in Pneumonia........ 90
Venery, excessive........ 145
Viburnum........ 55
Viburnum, opulus or cramp bark........ 348
Viburnum prunifolium........ 153
Vinegar in post partum hemmorrhage........ 312
Vomiting in pregnancy........107, 112. 302

W

Walker, B. M., M. D., cystic tumor........ 161
Walker, B. M., M. D. partial paralysis etc. from phimosis........ 332
Walking man........ 222
Warts........ 53
Whooping Cough........26, 220, 119, 309, 157
Wine of gentian........ 220
Wine in cappillary bronchitis........ 343
Womb, hemorrhage from........ 5
Worms........ 91
Wounds of scalp........ 158

Y

Yerba Ruma........ 124
Yellow wash........ 60
Yellow fever cause and treatment of........295, 289
Yellow fever........265, 325
Yellow fever, National Relief Association........ 351
Yellow fever, microscopical observations in........ 373
Yellow fever, new theory of its propagation........ 285

Z

Zinc, cyanide of........ 59
Zinc, glycerite of the hypophosphites of........ 315
Zinc, hypophosphite of........ 140
Zinc, chloride of, in ulcers of the leg........ 148
Zinc, oxide of, in diarrhœa........ 390

### *EDITORIAL AND MISCELLANEOUS.*

☞ All communications relating to the business of The Record, for the years 1877 and 1878, must be addressed to DR. R C. WORD, Managing Editor Southern Medical Record, Atlanta, Ga.
☞ Brief and practical communications are solicited on all subjects pertaining to medicine; also reports o cases in practice.
☞ Send money by check, postal order or registered letter.
☞ Write your name, post-office, county and State plainly.

To Our Subscribers.—Friends, in accordance with notice given in our December number, we have entered all paying subscribers upon our list for 1878, who have not notified us to discontinue. *Only three names* have so notified us, and two of these with expressions of regret, while many new subscribers are coming in.

We are encouraged—and enter upon our duties with renewed zeal, and determination to please and benefit the busy practitioner. We have, however, incurred much expense, and shall be much cramped in our efforts until subscriptions come in. We hope, therefore, our friends will make prompt remittances. In the meantime, accept our thanks for your patronage and confidence, and our greetings and kind wishes for the new year.

